# Innate lymphoid cells in early tumor development

**DOI:** 10.3389/fimmu.2022.948358

**Published:** 2022-08-12

**Authors:** Kathrin Warner, Maryam Ghaedi, Douglas C. Chung, Nicolas Jacquelot, Pamela S. Ohashi

**Affiliations:** ^1^ Princess Margaret Cancer Centre, University Health Network, University of Toronto, Toronto, ON, Canada; ^2^ Department of Immunology, University of Toronto, Toronto, ON, Canada

**Keywords:** innate lymphoid cell (ILC), tumor development, damage associate molecular pattern (DAMP), cytokines, carcinogenesis, immunosurveillance, tumor immunity

## Abstract

Innate and adaptive immune cells monitor, recognize, and eliminate transformed cells. Innate lymphoid cells (ILCs) are innate counterparts of T cells that play a key role in many facets of the immune response and have a profound impact on disease states, including cancer. ILCs regulate immune responses by responding and integrating a wide range of signals within the local microenvironment. As primarily tissue-resident cells, ILCs are ideally suited to sense malignant transformation and initiate anti-tumor immunity. However, as ILCs have been associated with anti-tumor and pro-tumor activities in established tumors, they could potentially have dual functions during carcinogenesis by promoting or suppressing the malignant outgrowth of premalignant lesions. Here we discuss emerging evidence that shows that ILCs can impact early tumor development by regulating immune responses against transformed cells, as well as the environmental cues that potentially induce ILC activation in premalignant lesions.

## 1 Introduction

Tumorigenesis is a complex, multistep process in which normal cells evolve progressively to a neoplastic state. Thus, tumors are often preceded by different stages of premalignant tissue changes, including hyperplasia, metaplasia, and dysplasia, which are linked to an increased cancer risk. The immune system can detect tissue changes and eliminate transformed cells in a process referred to as tumor immunosurveillance. The original cancer immunosurveillance hypothesis was formulated in the 1950s and described that adaptive lymphocytes reduce tumor growth in response to recognizing tumor antigens (1, 2). Since then, this theory has been refined and cancer immunosurveillance is now widely accepted as being part of the cancer immunoediting process, wherein the tumor-suppressive and tumor-promoting activities of the immune system shape tumor development. This process is divided into three different phases: elimination (cancer immunosurveillance), equilibrium (cancer persistence/dormancy), and escape (cancer progression) (3–5).

Our current understanding of cancer immunosurveillance is primarily based on studies in mice, which have shown that the immune system can prevent the outgrowth of many different types of primary and transplantable tumors (4). Evidence for the importance of the immune system in preventing tumor development in humans is found in studies showing increased incidences of malignancies in immunocompromised patients with AIDS and recipients of organ transplants using immunosuppressants, as well as spontaneously regressing benign and malignant melanocytic lesions accompanied by lymphocytic infiltrates (4). Despite the immune system’s anti-tumorigenic activities, deregulated inflammatory responses have also been linked to carcinogenesis and often precede tumor development (6). Thus, the immune system does not only protect the host against tumor development but also promotes progression of premalignant to malignant cells.

A comprehensive view of tumor immunosurveillance would include not only adaptive immune cells but also innate immune cells since it is well known that they detect and destroy transformed cells (4, 5). Besides T cells, natural killer (NK) cells are known to play a key role in cancer immunosurveillance (7). NK cells are innate lymphoid cells (ILCs) that mirror CD8^+^ T cytotoxic cells and secrete cytotoxic molecules such as granzymes and perforin to eliminate virus-infected cells and tumor cells. Increasing evidence suggests that other ILC family members also play an important role in the immune response against tumors (8) and their role in tumor development is starting to being explored. ILCs have been classified into NK cells, ILC1s, ILC2s, ILC3s, and lymphoid tissue inducer (LTi) cells based on their cytokine and transcription factor expression profiles, and developmental pathways (9). ILC1s, ILC2s and ILC3s share features with CD4 T helper (h)1, Th2, and Th17/22 subsets, respectively. NK cells and ILC1s express the transcription factor T-box transcription factor 21 (T-BET) and secrete interferon (IFN)-γ. In addition, NK cells, but not ILC1s, require the transcription factor Eomesodermin (EOMES) for their development. However, a proportion of ILC1s can express EOMES (10). ILC1s are involved in the immune response against viruses and intracellular bacteria. They express multiple granzyme molecules, but at lower levels compared to NK cells. ILC2s are dependent on the transcription factors GATA-binding protein 3 (GATA3) and retinoic acid-related orphan receptor (ROR)α and produce classical type 2 cytokines such as interleukin (IL)-4, IL-5, and IL-13 in response to parasite infection and allergen exposure. ILC3s and LTi cell subsets share a characteristic expression of the retinoic acid receptor-related orphan nuclear receptor γt (RORγt) and the cytokines IL-17A and IL-22 but follow different developmental pathways. ILC3s are immune effectors that contribute to host defense against extracellular bacteria and fungi, whereas LTi cells initiate the development of fetal lymphoid tissues (9).

NK cells circulate in the body, whereas the other ILC subsets are primarily tissue resident cells that preferentially reside in barrier tissues. In addition to providing immunity against infections, they also play critical roles in maintaining tissue homeostasis by responding rapidly to environmental cues, initiating effector responses in a tissue-specific manner and interacting with tissue-resident cells (11). This makes them ideally suited to sense malignant transformation and initiate anti-tumor immunity. However, ILCs have been associated with pro-tumor and anti-tumor activities in established tumors (8) and could therefore have a dual role during tumor development as well. In this review, we discuss the stress signals that could potentially activate ILCs during tumor development and recent advances supporting a role of ILCs in immune surveillance and carcinogenesis.

## 2 ILCs and tumor development

Premalignant lesions arise from various causes, including infection, inflammation, and environmental exposures. Innate immune cells are considered the first responders to cellular stress and mediate adaptive immune responses. This is supported by a study that profiled 122 bronchoscopy biopsies from 77 patients using gene-expression profiling and multispectral imaging, which included 9 morphological stages of invasive lung squamous cell carcinoma (SCC) development. During hyperplasia, the earliest stage of transformation, there was an increase of innate immune cells, such as neutrophils, activated mast cells and NK cells, and resting dendritic cells (DCs), as well as naïve CD4 T cells. This was followed by an increase of CD8 T cells and activated memory CD4 T cells in metaplastic and dysplastic tissues. Thus, NK cells are part of an early immune response against tissue changes associated with malignant transformation. Although this study did not assess other ILC populations in these tissues, it is likely that they respond to the same stress signals that activate other innate immune cells (section 3.).

Studies in mice have provided important evidence that ILCs play a role in mediating tumor immunosurveillance, either directly or indirectly through modulation of effector immune cell responses. Expression of IFN-γ and the effector molecule tumor necrosis factor (TNF)-related apoptosis-inducing ligand (TRAIL) by NK cells has been shown to prevent tumor initiation in mice (12, 13). In addition, low cytotoxic activity of NK cells is associated with an increased cancer risk in humans (14–16). Mice depleted of NK cells and ILC1s by anti-NK1.1 or anti-asialo-GM1 are more susceptible to the formation of chemically induced tumors (17). Furthermore, tumor incidence in Rag1^−/−^IL2Rγ^−/−^ and Rag2^−/−^IL2Rγ^−/−^ mice, which in addition to B and T cells also lack ILCs, was increased compared to Rag1^−/−^ and Rag2^−/−^ mice lacking only adaptive immune cells (18, 19).

**Figure 1:**
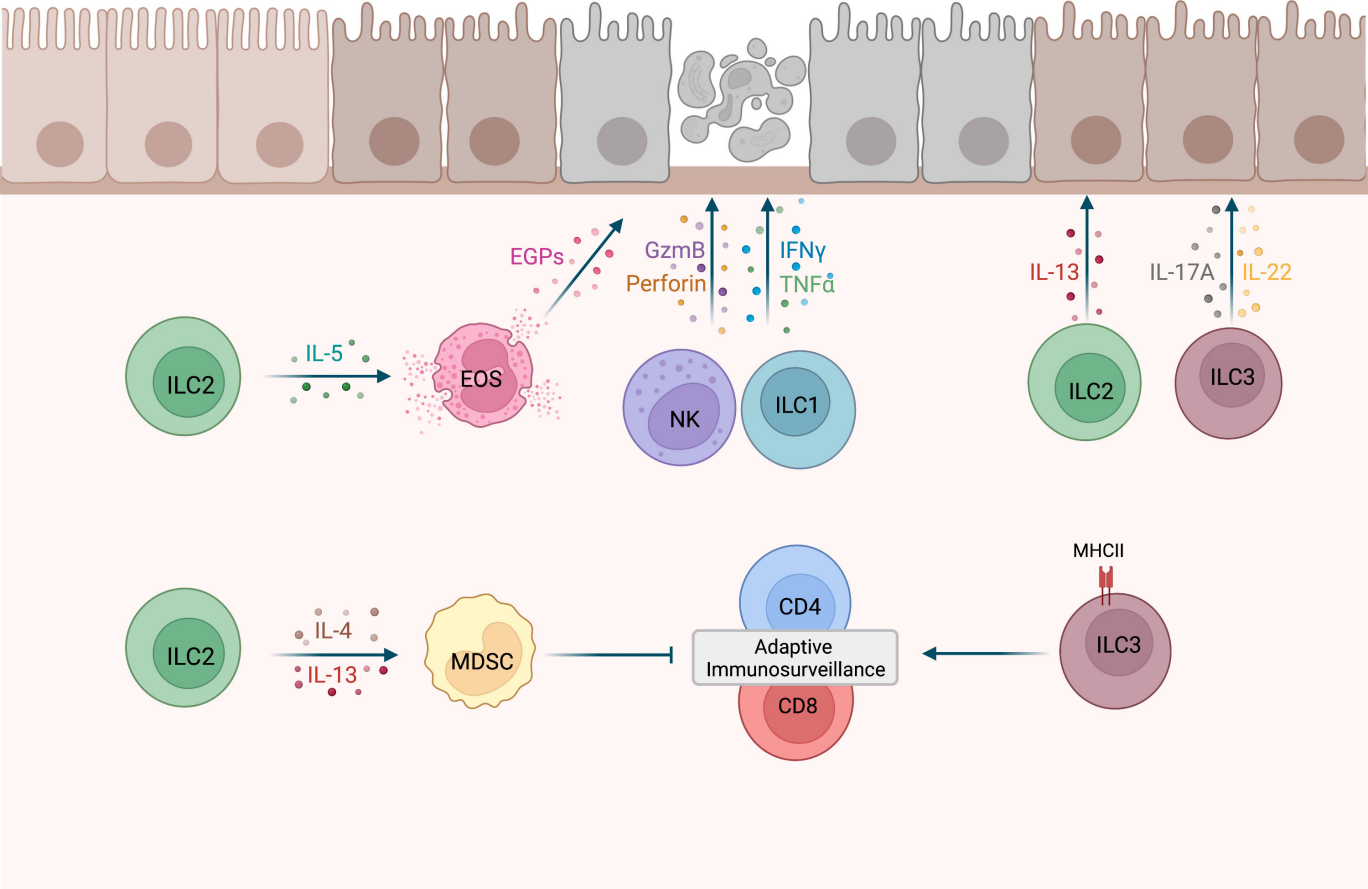
Potential roles of ILCs during tumor development. NK cells and ILC1s have shown cytotoxic activity against precancerous cells (12, 13, 20). IL-5-secreting ILC2s may recruit eosinophils (EOS) to precancerous tissues and activate cytotoxic effector functions in EOS, such as the release of eosinophilic granule proteins (EGPs) (21, 22). MHCII^+^ ILC3s were shown to promote CD4 and CD8 cell responses to prevent tumor development (23). Conversely, ILC2s may drive MDSCs activation and subsequent T cell suppression *via* IL-4 and IL-13 secretion (24), as well as metaplasia development *via* the release of IL-13 (25). IL-17A and IL-22 expression by ILC3s may promote clonal expansion of precancerous cells (26). This figure has been created with BioRender.com.

With the exception of NK cells, the contribution of individual ILC subsets to immune responses during early tumor development is less well defined. Using the MMTV-PyMT mammary tumor model, Dadi et al. demonstrated that a cytotoxic ILC1-like population accumulates in precancerous lesions (20). Importantly these cells were dependent on IL-15 and displayed toxicity against tumor cells (20) (**Figure 1**). Interestingly, unlike the ILC1-like population, NK cells did not expand in these precancerous lesions, suggesting that tissue-resident ILC1-like cells may play a more important role in early sensing of cellular transformation. ILC2 stimulation by epithelial and/or Th2-derived cytokines induces IL-5, GM-CSF and IL-13 expression, leading to eosinophil recruitment, activation and survival (21, 22). In a model for chemically-induced fibrosarcomas, IL-5 overexpression protected mice from tumor establishment through an increased recruitment of eosinophils to the tumor and surrounding connective tissue (27). Thus, ILC2s could potentially mediate tumor immunosurveillance by regulating eosinophil accumulation in premalignant tissues (**Figure 1**). A protective role of ILC2s during tumor development was also described in a chemically-induced colorectal cancer (CRC) mouse model as ILC2-deficient mice had an increased tumor burden compared to WT mice (28). This is further supported by a recent study showing that IL-33 mediated expansion of ILC2s was associated with reduced colonic inflammation in a colitis model (29). ILC2s may be involved in the immune response against developing CRC by activating eosinophils, as eosinophils have been shown to prevent the development of CRC in a colitis-associated cancer model independently of CD8^+^ T cells (30). However, in an adenomatous polyposis coli (*Apc*)-mutation-driven model of spontaneous intestinal tumorigenesis, IL-25 activated ILC2s promoted CRC development by promoting myeloid-derived suppressor cell (MDSCs) function to suppress T cell responses (24). In addition, another study found that in response to gastric tissue damage in mice, IL-13-secreting ILC2s are recruited to the gut mucosa and drive metaplasia development (25). Together, these studies provide evidence that ILC2s might promote malignant transformation, depending on the environmental cues and tissues involved (**Figure 1**). Additional studies will be required to distinguish between pro- and anti-tumorigenic functions of ILC2s, similar to what is observed for their role in established tumors (8).

A recent study by Goc et al. identified a major histocompatibility complex class II positive (MHCII+) ILC3 population in precancerous adenomas in mice and humans (23). In a spontaneous CRC mouse model, deletion of ILC3-specific MHCII resulted in an increased number of advanced tumors and a significant reduction in overall survival, suggesting that MHCII^+^ ILC3 limit tumor development. Further analysis revealed that mice lacking MHCII expression in ILC3s were characterized by a significant reduction of Th1 and T-bet^+^ CD8 T cells, thereby providing evidence that interactions between ILC3s and T cells promote type-1 immunity (**Figure 1**). Although this study supports a role of ILC3s in tumor immunosurveillance, there are other studies that implicate ILC3s in tumorigenesis. Dysregulated IL-23 mediated ILC3 activation and IL-17 and IL-22 production has been shown to promote gut inflammation and tumorigenesis (31, 32). In addition, in a hepatocellular carcinoma (HCC) mouse model established by a murine HCC cell line, IL-23 over-expression promoted HCC development in an IL-17-dependent manner (33). Interestingly, most IL-17-producing cells in early tumors were NCR^−^ILC3s, suggesting that they are the initial responders to IL-23. Using a model for UV–induced cutaneous carcinogenesis, Lewis et al. also demonstrated that chronic UV exposure leads to an increase in IL-22 and IL-17A-producing ILC3s in the skin, which drive mutant keratinocytes clonal expansion in the absence of T cells (26) (**Figure 1**). Based on studies in established tumors, it is known that ILC3s can have conflicting functions (8, 34) and current evidence suggests that this might also be the case for their role during early tumor development.

## 3 Stress signals during cell transformation and their potential role in ILC activation

Stressed and dying cells in precancerous tissues express and/or release various endogenous danger molecules, such as damage-associated molecular patterns (DAMPs), cell-surface receptors, and cytokines, that activate the immune system (35). Early detection of stress signals is important for successful cancer immunosurveillance. However, some signals may activate inflammatory responses that contribute to malignant transformation instead of protecting against it. ILCs sense changes in the tissue microenvironment through a broad array of cell surface and intracellular receptors, including costimulatory receptors, cytokine and chemokine receptors (36–38). Binding of ligands to these receptors also drives ILC plasticity, thereby shaping their function and phenotype (39). Here, we will discuss how known stress signals released by precancerous cells could potentially activate ILCs and initiate ILC-mediated immune responses that impact tumor development. In particular, we will focus on signals associated with an inflammatory response in the absence of infection. ILCs express toll-like receptors that could potentially recognize pathogen-associated molecular patterns (PAMPs) during infection-associated tumor development (40, 41). However, it is likely that the immune responses to these infections and developing tumors in the same tissues overlap and the role of ILCs in response to intracellular pathogens has already been extensively reviewed elsewhere (40).

### 3.1 DAMPs

DAMPs are endogenous danger signals released by damaged or dying cells to induce an immune response during non-infectious inflammation. Although DAMPs have been proposed to activate local antigen-presenting cells (APCs), it is also possible that these signals promote inflammation by activating ILCs. However, the role of inflammatory signals during tumor development is often not clearly defined as they can also be associated with cancer growth. Here, we discuss DAMPs with receptors found on ILCs (**Table 1**) and their potential role during tumor initiation and activation of ILC responses.

**Table 1:** Expression of DAMPs and cytokine receptors by ILCs.

DAMP/Cytokine	Receptor(s)	NK cells		ILC1		ILC2		ILC3		Reference(s)
		Ms	Hu	Ms	Hu	Ms	Hu	Ms	Hu	
**HMGB1**	RAGE	Yes	^nd^	^nd^	^nd^	Yes	^nd^	^nd^	^nd^	(42, 43)
	TLR2	Yes	Yes	^nd^	Yes	Yes	Yes	^nd^	Yes	(44–48)
	TLR4	Yes	Yes	^nd^	Yes	Yes	Yes	^nd^	Yes	(44, 47–49)
**ATP**	*P2Y_1/2/4/6/11-14_	Yes	Yes	^nd^	^nd^	^nd^	Yes	^nd^	Yes	(50–53)
	*P2X1-7	Yes	Yes	Yes	^nd^	^nd^	^nd^	^nd^	Yes	(51, 54–56)
**IL-33**	ST2	Yes	Yes	^nd^	^nd^	Yes	Yes	^nd^	^nd^	(57–60)
**IL-25**	IL-25R	No	No	^nd^	No	Yes	Yes	No	No	(9, 61)
**IL-12**	IL-12R	Yes	Yes	Yes	Yes	Yes	Yes	Yes	^nd^	(36, 62–66)
**IL-15**	IL-2Rβ	Yes	Yes	Yes	^nd^	Yes	Yes	Yes	Yes	(20, 67–71)
**IL-18**	IL-18R	Yes	Yes	Yes	Yes	Yes	Yes	^nd^	Yes	(62, 71, 72)
**IL-23**	IL-23R	No	Yes	No	Yes	^nd^	No	Yes	Yes	(9, 36, 73)

Mouse, Ms; Human, Hu; nd, not determined. *Expression of 1 or more of indicated receptors has been reported.

#### 3.1.1 High mobility group box 1

HMGB1 is a nuclear protein widely expressed in mammalian cells and is involved in various cellular processes, including the maintenance of chromosome structure and function, DNA damage repair and transcription. Cells undergoing necrosis passively release HMGB1, while various exogenous and endogenous stimuli can induce the active release of HMGB1 by immune cells, endothelial and epithelial cells (74). Notably, HMGB1 is one of the DAMPs released during immunogenic cell death, which is induced by infectious pathogens and anticancer chemotherapeutics (75). Extracellular HMGB1 acts as a danger signal that mediates inflammation and repair responses *via* binding to the inflammatory receptor advanced glycation end-products (RAGE) and Toll-like receptors (TLRs). These receptors are expressed by various immune cells, including NK cells and ILC2s (**Table 1**). In established tumors, conflicting roles have been described for HMGB1, including the activation of tumor-promoting inflammatory responses and immunosuppressive pathways, as well as the induction of anti-tumor responses (76). Current evidence supports a pro-tumorigenic role of HMGB1 during tumor development. Studies assessing serum levels in patients with normal tissue, premalignant lesions, early and advanced stages of cancer, showed that HMGB1 levels increase according to the progression of gastric and hepatocellular carcinogenesis (77, 78). This suggests that HMGB1 is released during cellular transformation. However, HMGB1 might promote tumor development rather than activating immune responses against premalignant cells as chemically-induced skin and inflammation-induced liver cancer development was inhibited in mice deficient for the HMGB1 receptor RAGE (79, 80). This is further supported by a study of premalignant and malignant lesions of the uterine cervix, which showed that HMGB1 inhibited maturation of plasmacytoid DCs to render them tolerogenic (81). More studies are needed to understand the complex role of HMGB1 during tumor development.

The impact of HMGB1 expression on ILCs in premalignant lesions has not been assessed yet. However, studies in established tumors support a role of HMGB1 in NK activation. In mice, HMGB1 released from chemotherapy-induced necrotic tumor cells induced NK cell activation and infiltration into the tumor (82). Mouse and human ILC2s were also shown to express RAGE and respond to HMGB1 activation (42, 83). Thus, ILC2s could potentially respond to HMGB1 *via* RAGE or its other receptors (**Table 1**) in premalignant and malignant lesions, however, if and what effect this has on tumor development is currently unknown.

#### 3.1.2 Adenosine triphosphate

ATP is a multifunctional nucleotide best known for storing and transferring energy in cells. Extracellular ATP is actively secreted by stressed cells or passively released by dead cells, and acts on P2 purinergic receptors (**Table 1**). Released ATP is enzymatically converted into adenosine by the ectonucleotidases CD39 and CD73, which binds to P1 purinergic receptors. Purigeneric receptors are widely expressed by various immune and non-immune cells. Established tumors are characterized by high concentrations of ATP and adenosine. Adenosine and ectonucleotidases are predominantly associated with tumor-promoting and immunosuppressive activities (84). Extracellular ATP-binding can support or inhibit anti-tumor responses, depending on ATP concentration, the type of receptor, and the target cell (85). The role of extracellular ATP and adenosine in tumor development has not been extensively studied and related studies have provided contradictory results. For example, studies assessing the role of the ATP receptor P2X7R in inflammation-associated CRC models have described an increase as well a reduction of tumor incidence in mice deficient for P2X7R (86, 87). Evidence for the involvement of ATP and adenosine in activating ILC responses was provided in the context of tissue repair and inflammation. Blocking of the ATP receptor P2X1R abrogated cytokine secretion in NK cells and ILC1s and impaired liver regeneration in a model for partial hepatectomy (54). In a chemically induced intestinal injury model, IL-22-secreting ILC3s accumulated in the colon and were important for the control of colitis. Treatment with an ectonucleotidase inhibitor prevented ILC3 activation and IL-22 production by ILC3s. Thus, accumulation of ATP was associated with ILC3 inhibition, while conversion to adenosine lead to activation of ILC3s (88).

#### 3.1.3 IL-33

IL-33 acts as a cytokine and a DAMP, as it’s released by epithelial cells, endothelial cells, and fibroblasts in response to tissue damage, as well as actively secreted by APCs. The primary receptor for IL-33 is ST2, which exists in soluble form as a decoy receptor, and as part of a membrane-bound heterodimer together with the co-receptor IL-1 receptor accessory protein (IL1RAP) that initiates downstream signaling (89). IL-33 activates ST2-expressing mast cells, eosinophils, macrophages, ILC2s, NK cells, and T cell subsets, such as Th1, Th2, CD8^+^ T cells, and Tregs (90) (**Table 1**), thereby modulating both innate and adaptive immune responses. IL-12-induced IFN-γ production by murine and human NK cells is enhanced by IL-33 (57, 58) and as a central regulator of type 2 immunity, IL-33 mediates ILC2 activation and proliferation (9). IL-33 has a dual role in established cancer and has been associated with both anti-tumor and pro-tumor immune responses (91). Anti-tumor functions are mostly attributed to the induction of type 1 immune responses and pro-tumor activities include the activation of Tregs and type 2 responses. The role of IL-33 in tumor development has been mostly studied in the context of CRC and current data suggests that IL-33 can contribute to the pathogenesis (92, 93) as well as the suppression of CRC development (94). IHC analysis of precancerous colorectal lesions also revealed that precancerous epithelial cells, as well as stromal and endothelial cells can be a source of IL-33 (95). It remains to be elucidated how IL-33 contributes to the described pro- or anti-tumorigenic functions of ILC2s in CRC and other cancers, and if the cytokine milieu in these tissues allows for IL-33-mediated enhancement of NK cell responses.

#### 3.1.4 IL-25

Like IL-33, IL-25, also known as IL-17E, functions as a cytokine and a DAMP. IL-25 signals through the IL-25R, a heterodimer complex composed of IL-17RB and IL-17RA, and is produced by epithelial cells and immune cells including activated Th2 cells, mast cells, and eosinophils. Expression of IL-25 is regulated by harmful environmental cues and plays an important role in activating Th2 immune responses. Dysregulated IL-25 expression has been linked to airway inflammation and severe asthma exacerbation (96). IL-25 has also been shown to promote inflammatory responses in the context of colitis (97), suggesting that it might favor tumor development. However, pro- and anti-tumorigenic functions have been described for IL-25. A study by Thelen et al. found that blocking of IL-25 in a colitis-driven colon cancer model, leads to increased tumor burden and a decrease of eosinophils in colon tissues (98). Conversely, Jou et al. found that IL-25 treatment of *Apc* ^1322T/+^ mice, an APC-mutation-driven CRC mouse model, resulted in an increased tumor burden, which was accompanied by increased ILC2 infiltration (24). In this model, ILC2 indirectly suppressed anti-tumor T cell responses by activating MDSCs *via* IL-4 and IL-13. Genetic ablation of ILC2s or IL-25, or treatment with IL-25 blocking antibodies in these mice led to reduced tumor growth and increased survival.

### 3.2 Cytokine-mediated ILC activation

Cytokines are small soluble proteins that are crucial for immune cell homeostasis and the regulation of innate and adaptive immune responses. Proinflammatory cytokines are released in response to cellular stress and infection to alert the immune system to the presence of potential danger. Transformed cells are known to secrete and promote production of diverse cytokines in different types and stages of cancers (99). Furthermore, DCs and macrophages that are activated in response to cellular stress also start expressing proinflammatory cytokines and contribute to the local cytokine milieu. Studies of murine and human tissues found that there is a reduction of proinflammatory cytokines when premalignant lesions or early tumors progress to clinically apparent tumors (100–102) and an increase of immunosuppressive cytokines (103). In addition, aberrant release of proinflammatory cytokines contributes to tumor progression and immune cell dysfunction. Thus, the cytokines present in premalignant tissues will shape local immune responses, including ILC activity. Common cytokines associated with ILC activation are IL-12, IL-15 and IL-18 for ILC1s and NK cells; IL-2, IL-18, IL-25, IL-33, and thymic stromal lymphopoietin (TSLP) for ILC2s; and IL-1ß and IL-23 for ILC3s (104). Here, we discuss the role of ILC-activating cytokines in the context of tumor development.

#### 3.2.1 IL-12

The heterodimeric pro-inflammatory cytokine IL-12 is known for its role in activating anti-tumor immunity (105). IL-12 is produced by APCs, such as DCs and macrophages, and induces Th1 differentiation and the production of IFN-γ in T and NK cells (106). In addition, IL-12 negatively regulates Treg cell function and proliferation (107, 108), as well as Th2 and Th17 differentiation (106). The lack of IL-12 subunits p35 or p40 results in increased or earlier tumor development in mice (109–112). These studies highlight the importance of IL-12 in regulating early immune responses against transformed cells. In addition, various IL-12 gene polymorphisms leading to decreased IL-12 production are associated with increased susceptibility to cancer (113). Besides NK cells, ILC1 also respond to IL-12 stimulation and IL-12 promotes conversion of ILC2s and ILC3s to IFN-γ-producing ILC1s (114).

#### 3.2.2 IL-15

IL-15 is a proinflammatory cytokine crucial for the proliferation and survival of T cells and NK cells (115). Lack of IL-15 in mice results in severe reduction of both cell types (116). IL-15 mainly exists as a heterodimeric complex with membrane bound or the soluble form (sIL-15) of IL-15Rα, and binds to the IL-2Rβγ heterodimer on nearby effector cells. Cellular sources of IL-15 include monocytes, macrophages, DCs, stromal cells, and epithelial cells (115). Various murine tumor cell lines have also been shown to express IL-15 (102). IL-15 enhances anti-tumor responses of murine and human CD8^+^ T cells and NK cells (117–120), and is considered a promising agent for cancer immunotherapy (121). In NK cells, IL-15 treatment leads to upregulated expression of NKG2D and the cytotoxic effector molecules TRAIL and perforin (122). In transplanted and spontaneous tumor models, IL-15-deficiency and the subsequent reduction in T and NK cell numbers leads to accelerated tumor development (102, 123–125), suggesting that IL-15 plays a critical role during early anti-tumor responses. Moreover, deletion of IL-15 in CRC patients was associated with a higher risk of relapse and reduced disease-free survival (126). Besides NK cells, mouse and human helper ILC1s have also been shown to respond to IL-15 (20, 67, 127–129). Other ILC populations may also get activated in response to IL-15 in early tumors as IL-15 has been shown to induce conversion of ILC3s into IFN-γ-producing ILC1s and cytotoxic NK cells (114, 130).

#### 3.2.3 IL-18

IL-18, originally termed IFN-γ-inducing factor, is part of the IL-1 family. Binding of IL-18 to its receptor, which consists of IL-18Rα and IL-18Rβ, can be prevented by the soluble IL-18 binding protein (IL-18BP). IL-18 is expressed by various types of cells, including macrophages, DCs, and epithelial cells (131). Together with IL-12, IL-18 induces Th1 responses by acting on T cells and NK cells to induce IFN-γ production. Treatment with IL-18 also enhances Fas-L-expression and FAS-L-mediated cytotoxicity in NK cells and CD8^+^ T cells (132, 133). In patients with cervical premalignant lesions, low expression of IL-18 was associated with an increased risk of progression of pre-neoplastic lesions to cancer (134), supporting its role in activating immune responses against transformed cells.

#### 3.2.4 IL-23

IL-23 is an IL-12 family member and a heterodimer that consists of a p19 and a p40 subunit, which is shared with IL-12. The IL-23 receptor is made up by IL-23R and IL-12Rβ1 subunits. The main sources for IL-23 are macrophages and DCs, which release IL-23 in response to exogenous or endogenous signals associated with host defense and wound healing (135). IL-23 plays a crucial role in the differentiation and maintenance of Th17 cells, and promotes Th17 production of IL-17A, IL-17F, IL-6, IL-22, and TNF-α. IL-23 is also one of the main mediators of ILC3 activation, resulting in their constitutive secretion of IL-22, which in turn acts on mucosal epithelium to induce the expression of antimicrobial peptides, tight-junctions and promote the colonization of beneficial commensal bacteria protecting against intestinal inflammation (136). The role of IL-23 in cancer is complex and has been associated with tumor-promoting and tumor-suppressive activities (135). Its role in tumor development is not well understood. In a model for MCA-induced fibrosarcomas, tumor incidence was reduced in mice deficient for the IL-23 subunit p19 and depletion of NK cells, but not CD8^+^ T cells, abrogated the protective effect of IL-23 depletion (137). Conflicting roles were described for IL-23 in the development of chemically-induced cutaneous tumors, as tumor growth was either inhibited (112) or enhanced (138) in p19-deficient mice, depending on the background strain. A study of murine and human premalignant lesions for head and neck squamous cell carcinoma reported elevated levels of IL-2, IFN-γ, TNF-α, IL-6, and IL-17 in premalignant lesions, which was dependent on IL-23 and accompanied by an increase in IFN-γ^+^ CD4^+^ T cells (100, 139). In IL-23R KO mice, production of these cytokines was reduced and the progression of premalignant oral lesions toward cancer accelerated (139), suggesting that IL-23 has a protective role during tumor development. Although IL-23–driven immune responses have been primarily linked to T cells, IL-23 could potentially activate ILC3 in premalignant lesions as well.

### 3.3 Cell surface receptors and molecules

Unlike T and B cells, ILCs do not express antigen receptors and therefore do not recognize specific tumor antigens. However, ILCs express other activating cell surface receptors that initiate anti-tumor responses (37). NK cell activity is regulated by a balance between various activating and inhibitory receptors that bind to cognate ligands on target cells (140, 141). Healthy cells express MHCI molecules on their surface that act as inhibitory ligands for inhibitory receptors on NK cells, such as killer cell immunoglobulin-like receptors (KIRs) and the CD94/NKG2A heterodimer, thereby contributing to tolerance from NK cell recognition (142). Other central activating and co-activating NK cell receptors include the natural cytotoxicity receptors (NCRs) NKp30, NKp44, and NKp46, CD16, NKG2D, NKG2C, DNAX Accessory Molecule-1 (DNAM-1), and 2B4 (142, 143). NK cell activating ligands are often upregulated in response to cellular stress associated with infection and malignant transformation (144).

The NKG2D receptor recognizes several MHCI-like ligands, including MHCI-polypeptide-related sequence MICA, MICB, and UL16 binding proteins (ULBP1-6) in humans, and retinoic acid early inducible-1 family (RAE-1α-ϵ), H60a-c, and murine UL16 binding protein-like transcript (MULT-1) in mice (145). Homodimerization of NKG2D by membrane-expressed ligands recruits phosphatidylinositol 3-kinase (PI3K) and growth factor receptor-bound protein 2 (GRB2), resulting in a phosphorylation cascade. If then, the overall balance of signaling from both activating and inhibitory receptors favors NK cell activation, it can stimulate NK cell effector functions resulting in perforin/granzyme-mediated cytotoxicity and cytokine release. NKG2D is considered an important receptor in NK cell immune surveillance of cancer since spontaneous tumor development was shown to be more frequent in NKG2D-deficient mice compared to wild type mice (146). Cell surface expression of NKG2D ligands is low or not present on healthy tissues, but is upregulated on rapidly proliferating cells, virally infected cells, and cancer cells (147–150). Ectopic expression of NKG2D ligands in tumor cell lines results in tumor cell rejection in mice (151, 152). However, only a few studies have examined the expressionof these ligands in premalignant tissue. In mouse models for cutaneous carcinogenesis, exposure to carcinogens induces the expression of NKG2D ligands in skin cells (153–155). NK cell depletion in one of these studies resulted in higher numbers of papillomas (153), suggesting that NK cells play an important role in the elimination of DNA-damaged skin cells. Notably, recruitment of NK cells to the epidermis was dependent on TNF-α-induced chemokines CCL2 and CXCL10. In humans, premalignant skin lesions lacked expression of MICA (156) and low expression of MICA, MICB, and ULBP1 is found on thymic hyperplasia (157). Further studies are needed to understand the role of NKG2D ligands during tumor development in humans.

The activating receptor DNAM-1 is expressed by many lymphocyte subsets, including NK cells and T cells. Binding of DNAM-1 (CD226) to its ligands PVR (CD155) and Nectin-2 (CD112) induces NK cell cytotoxicity (158). These ligands are highly expressed on tumor cells, but only low or no expression is found on healthy tissues. Lack of DNAM-1 expression results in reduced T and NK cell cytotoxicity against tumor cells and accelerated tumor outgrowth of chemically-induced fibrosarcomas (159) as well as spontaneous tumors (160, 161). PVR and Nectin-2 overexpression was observed in human premalignant lesions of CRC and pancreatic ductal adenocarcinoma, respectively (162, 163). Together, these studies provide evidence for a role of DNAM-1 in tumor immune surveillance, which likely not only involves NK cell but also T cell activation. Notably, DNAM-1 is also expressed by human peripheral blood ILC2s (164) and murine liver ILC1s (55). DNAM-1-mediated ILC1 activation was critical for their activation and production of IFN-γ. Thus, DNAM-1-ligands expressed on premalignant and malignant tissues may also activate DNAM-1-expressing non-NK cell ILCs.

NCR receptors were originally identified based on their ability to mediate cytotoxic functions of NK cells. The three known NCRs, NKp46, NKp44, and NKp30, comprise a family of type I transmembrane (TM) receptors and are encoded by the genes, *NCR1, NCR2*, and *NCR3*, respectively (165). Originally, these receptors were thought to be NK cell specific surface molecules, but many studies have provided evidence for expression on other cell types, including a subset of T cells, ILC1s and ILC3s (166–168). In the context of cancer, NCRs bind to a broad range of soluble, membrane-bound and nuclear ligands, including B7H6, platelet-derived growth factor (PDGF)-DD, and Galectin-3. However, the full spectrum of NCR ligands and their role in cancer remains to be fully characterized. Studies have shown that NKp46 is required for expression of the apoptosis-inducing ligand TRAIL on NK cells and ILC1s in mice, and genetic deficiency of NKp46 impairs tumor clearance (169–174), thereby implicating a role for NKp46-mediated activation of NK cells and ILC1s in tumor immunosurveillance. In addition, expression of the NKp30 ligands B7H6 and BAT3 by tumor cells was shown to trigger NK-cell cytotoxicity and cytokine secretion (175–177). The expression of NKp46 and NKp30 in human precancerous lesions is variable. NKp46 ligands were shown to be expressed on human benign and malignant melanocytic lesions (178), but NKp46 and NKp30 ligands were only found on primary human prostate tumors and not benign prostate hyperplasia (179). B7H6 was expressed in high-grade but not low-grade cervical lesions (180). Thus, it remains to be determined during which stage of tumor development NCR ligands mediate NK cell responses against tissue changes associated with malignant transformation.

In addition, expression of NKp30 and NKp44 was also reported on tumor-associated ILC2s and ILC3s, respectively (167, 181). In these studies, ILC3s were shown to interact with tumor cells and tumor-associated fibroblasts *via* NKp44, and were associated with a protective role against cancer, whereas NKp30^+^ ILC2s interacted with tumors cells *via* B7H6 and promoted an immunosuppressive tumor microenvironment. Further studies are needed to decipher the role of NCRs on helper ILCs during early tumor immunosurveillance.

## 4 Perspectives

Despite the growing body of research on ILCs, there is still a lot we do not understand about how the responses of these primarily tissue-resident cells are shaped by disease- and tissue-specific signals. This incomplete knowledge is reflected by research studies describing conflicting roles for ILCs in inflammation, immunopathological conditions, and cancer. This review specifically highlights a gap in our understanding of the role of ILCs in immunosurveillance and carcinogenesis. Our current knowledge on ILCs in cancer is mostly based on studies in established tumors. However, early tumors and premalignant tissues are characterized by a different tissue environment than established tumors. This has a profound impact on ILC responses as these cells sense a large variety of tissue signals, which modulate their phenotype and function. Signals that could potentially activate ILC-responses at the pretumor stage are highlighted in this review and could serve as a starting point for future studies. In particular, studies in premalignant tissues of patients are needed to improve our understanding of the precancerous tissue microenvironment and the early immune responses against malignant transformation. It also remains to be determined if and to what extent signals found in the established tumor microenvironment, such as lactic acid and hypoxia (182, 183), shape ILC functions in precancerous lesions. A better understanding of ILC responses in early tumor development will also provide novel insights regarding the overall regulation of ILC responses in response to cellular stress.

## Author contributions

Conceptualization, data curation, writing—original draft preparation, KW; Figure design, KW; writing – reviewing and editing, KW, MG, DC, NJ, and PO; Supervision, project administration, funding acquisition, PO. All authors have read and agreed to the published version of the manuscript.

## Funding

This work is funded by the Canadian Institutes for Health Research (CIHR FDN #143220) and the Canadian Cancer Society in memory of Mr. JIM JoTak (CCS grant #706152).

## Conflict of interest

The authors declare that the research was conducted in the absence of any commercial or financial relationships that could be construed as a potential conflict of interest.

## Publisher’s note

All claims expressed in this article are solely those of the authors and do not necessarily represent those of their affiliated organizations, or those of the publisher, the editors and the reviewers. Any product that may be evaluated in this article, or claim that may be made by its manufacturer, is not guaranteed or endorsed by the publisher.

## References

[1] Burnet M. Cancer–a biological approach: III. viruses associated with neoplastic conditions. IV. practical applications. BMJ (1957) 1:841–7. doi: 10.1136/bmj.1.5023.841 PMC1973618 13413231

[2] Thomas L. Delayed hypersensitivity in health and disease. In: Lawrence H, editor. Cellular and humoral aspects of the hypersensitive states. New York: Hoeber-Harper. (1959) p. 529–32.

[3] Dunn GP Bruce AT Ikeda H Old LJ Schreiber RD. Cancer immunoediting: from immunosurveillance to tumor escape. Nat Immunol (2002) 3:991–8. doi: 10.1038/ni1102-991 12407406

[4] Vesely MD Kershaw MH Schreiber RD Smyth MJ. Natural innate and adaptive immunity to cancer. Annu Rev Immunol (2011) 29:235–71. doi: 10.1146/annurev-immunol-031210-101324 21219185

[5] Teng MWL Galon J Fridman W-H Smyth MJ. From mice to humans: developments in cancer immunoediting. J Clin Invest (2015) 125:3338–46. doi: 10.1172/JCI80004 PMC4588291 26241053

[6] Zhao H Wu L Yan G Chen Y Zhou M Wu Y. Inflammation and tumor progression: signaling pathways and targeted intervention. Signal Transduct Target Ther (2021) 6:263. doi: 10.1038/s41392-021-00658-5 34248142 PMC8273155

[7] Marcus A Gowen BG Thompson TW Iannello A Ardolino M Deng W. Recognition of tumors by the innate immune system and natural killer cells. Adv Immunol (2014) 122:91–128. doi: 10.1016/B978-0-12-800267-4.00003-1 24507156 PMC4228931

[8] Jacquelot N Seillet C Vivier E Belz GT. Innate lymphoid cells and cancer. Nat Immunol (2022) 23:371–9. doi: 10.1038/s41590-022-01127-z 35228695

[9] Vivier E Artis D Colonna M Diefenbach A Di Santo JP Eberl G. Innate lymphoid cells: 10 years on. Cell (2018) 174:1054–66. doi: 10.1016/j.cell.2018.07.017 30142344

[10] Meininger I Carrasco A Rao A Soini T Kokkinou E Mjösberg J. Tissue-specific features of innate lymphoid cells. Trends Immunol (2020) 41:902–17. doi: 10.1016/j.it.2020.08.009 32917510

[11] Murphy JM Ngai L Mortha A Crome SQ. Tissue-dependent adaptations and functions of innate lymphoid cells. Front Immunol (2022) 13:836999. doi: 10.3389/fimmu.2022.836999 35359972 PMC8960279

[12] Cretney E Takeda K Yagita H Glaccum M Peschon JJ Smyth MJ. Increased susceptibility to tumor initiation and metastasis in TNF-related apoptosis-inducing ligand-deficient mice. J Immunol (2002) 168:1356–61. doi: 10.4049/jimmunol.168.3.1356 11801676

[13] Takeda K Smyth MJ Cretney E Hayakawa Y Kayagaki N Yagita H. Critical role for tumor necrosis factor-related apoptosis-inducing ligand in immune surveillance against tumor development. J Exp Med (2002) 195:161–9. doi: 10.1084/jem.20011171 PMC2193611 11805143

[14] Hersey P Edwards A Honeyman M McCarthy WH. Low natural-killer-cell activity in familial melanoma patients and their relatives. Br J Cancer (1979) 40:113–22. doi: 10.1038/bjc.1979.147 PMC2009943 314301

[15] Strayer DR Carter WA Mayberry SD Pequignot E Brodsky I. Low natural cytotoxicity of peripheral blood mononuclear cells in individuals with high familial incidences of cancer. Cancer Res (1984) 44:370–4. 6690050

[16] Imai K Matsuyama S Miyake S Suga K Nakachi K. Natural cytotoxic activity of peripheral-blood lymphocytes and cancer incidence: an 11-year follow-up study of a general population. Lancet (2000) 356:1795–9. doi: 10.1016/S0140-6736(00)03231-1 11117911

[17] Smyth MJ Crowe NY Godfrey DI. NK cells and NKT cells collaborate in host protection from methylcholanthrene-induced fibrosarcoma. Int Immunol (2001) 13:459–63. doi: 10.1093/intimm/13.4.459 11282985

[18] O’Sullivan T Saddawi-Konefka R Vermi W Koebel CM Arthur C White JM. Cancer immunoediting by the innate immune system in the absence of adaptive immunity. J Exp Med (2012) 209:1869–82. doi: 10.1084/jem.20112738 PMC3457735 22927549

[19] Kubick BJ Fan X Crouch A McCarthy R Roop DR. Tracing the equilibrium phase of cancer immunoediting in epidermal neoplasms *via* longitudinal intravital imaging. J Invest Dermatol (2020) 140:891–900.e10. doi: 10.1016/j.jid.2019.08.446 31542435 PMC7080593

[20] Dadi S Chhangawala S Whitlock BM Franklin RA Luo CT Oh SA. Cancer immunosurveillance by tissue-resident innate lymphoid cells and innate-like T cells. Cell (2016) 164:365–77. doi: 10.1016/j.cell.2016.01.002 PMC4733424 26806130

[21] Nussbaum JC Van Dyken SJ von Moltke J Cheng LE Mohapatra A Molofsky AB. Type 2 innate lymphoid cells control eosinophil homeostasis. Nature (2013) 502:245–8. doi: 10.1038/nature12526 PMC3795960 24037376

[22] Jacquelot N Seillet C Wang M Pizzolla A Liao Y Hediyeh-Zadeh S. Blockade of the co-inhibitory molecule PD-1 unleashes ILC2-dependent antitumor immunity in melanoma. Nat Immunol (2021) 22:851–64. doi: 10.1038/s41590-021-00943-z PMC7611091 34099918

[23] Goc J Lv M Bessman NJ Flamar AL Sahota S Suzuki H. Dysregulation of ILC3s unleashes progression and immunotherapy resistance in colon cancer. Cell (2021) 184:5015–30.e16. doi: 10.1016/j.cell.2021.07.029 34407392 PMC8454863

[24] Jou E Rodriguez-Rodriguez N Ferreira A-CF Jolin HE Clark PA Sawmynaden K. An innate IL-25-ILC2-MDSC axis creates a cancer-permissive microenvironment for apc mutation-driven intestinal tumorigenesis. Sci Immunol (2022) 7:eabn0175. doi: 10.1126/sciimmunol.abn0175 35658010 PMC7612821

[25] Meyer AR Engevik AC Madorsky T Belmont E Stier MT Norlander AE. Group 2 innate lymphoid cells coordinate damage response in the stomach. Gastroenterology (2020) 159:2077–91.e8. doi: 10.1053/j.gastro.2020.08.051 32891625 PMC7726005

[26] Lewis JM Monico PF Mirza FN Xu S Yumeen S Turban JL. Chronic UV radiation–induced RORγt+ IL-22–producing lymphoid cells are associated with mutant KC clonal expansion. Proc Natl Acad Sci (2021) 118. doi: 10.1073/pnas.2016963118 PMC8449378 34504008

[27] Simson L Ellyard JI Dent LA Matthaei KI Rothenberg ME Foster PS. Regulation of carcinogenesis by IL-5 and CCL11: A potential role for eosinophils in tumor immune surveillance. J Immunol (2007) 178:4222–9. doi: 10.4049/jimmunol.178.7.4222 17371978

[28] Huang Q Jacquelot N Preaudet A Hediyeh-Zadeh S Souza-Fonseca-Guimaraes F McKenzie ANJ. Type 2 innate lymphoid cells protect against colorectal cancer progression and predict improved patient survival. Cancers (Basel) (2021) 13:559. doi: 10.3390/cancers13030559 33535624 PMC7867134

[29] Ngo Thi Phuong N Palmieri V Adamczyk A Klopfleisch R Langhorst J Hansen W. IL-33 drives expansion of type 2 innate lymphoid cells and regulatory T cells and protects mice from severe, acute colitis. Front Immunol (2021) 12:669787. doi: 10.3389/fimmu.2021.669787 34335571 PMC8320374

[30] Reichman H Itan M Rozenberg P Yarmolovski T Brazowski E Varol C. Activated eosinophils exert antitumorigenic activities in colorectal cancer. Cancer Immunol Res (2019) 7:388–400. doi: 10.1158/2326-6066.CIR-18-0494 30665890

[31] Chan IH Jain R Tessmer MS Gorman D Mangadu R Sathe M. Interleukin-23 is sufficient to induce rapid *de novo* gut tumorigenesis, independent of carcinogens, through activation of innate lymphoid cells. Mucosal Immunol (2014) 7:842–56. doi: 10.1038/mi.2013.101 24280935

[32] Kirchberger S Royston DJ Boulard O Thornton E Franchini F Szabady RL. Innate lymphoid cells sustain colon cancer through production of interleukin-22 in a mouse model. J Exp Med (2013) 210:917–31. doi: 10.1084/jem.20122308 PMC3646494 23589566

[33] Liu Y Song Y Lin D Lei L Mei Y Jin Z. NCR- group 3 innate lymphoid cells orchestrate IL-23/IL-17 axis to promote hepatocellular carcinoma development. EBioMedicine (2019) 41:333–44. doi: 10.1016/j.ebiom.2019.02.050 PMC6443584 30827928

[34] Bruchard M Geindreau M Perrichet A Truntzer C Ballot E Boidot R. Recruitment and activation of type 3 innate lymphoid cells promote antitumor immune responses. Nat Immunol (2022) 23:262–74. doi: 10.1038/s41590-021-01120-y 35102345

[35] Kroemer G Galassi C Zitvogel L Galluzzi L. Immunogenic cell stress and death. Nat Immunol (2022) 23:487–500. doi: 10.1038/s41590-022-01132-2 35145297

[36] Chiossone L Dumas P-Y Vienne M Vivier E. Natural killer cells and other innate lymphoid cells in cancer. Nat Rev Immunol (2018) 18:671–88. doi: 10.1038/s41577-018-0061-z 30209347

[37] Klose CSN Artis D. Innate lymphoid cells control signaling circuits to regulate tissue-specific immunity. Cell Res (2020) 30:475–91. doi: 10.1038/s41422-020-0323-8 PMC7264134 32376911

[38] Jacquelot N Ghaedi M Warner K Chung DC Crome SQ Ohashi PS. Immune checkpoints and innate lymphoid cells–new avenues for cancer immunotherapy. Cancers (Basel) (2021) 13:5967. doi: 10.3390/cancers13235967 34885076 PMC8657134

[39] Bal SM Golebski K Spits H. Plasticity of innate lymphoid cell subsets. Nat Rev Immunol (2020) 20:552–65. doi: 10.1038/s41577-020-0282-9 32107466

[40] Korchagina AA Koroleva E Tumanov AV. Innate lymphoid cells in response to intracellular pathogens: Protection versus immunopathology. Front Cell Infect Microbiol (2021) 11:775554. doi: 10.3389/fcimb.2021.775554 34938670 PMC8685334

[41] Sivori S Carlomagno S Pesce S Moretta A Vitale M Marcenaro E. TLR/NCR/KIR: Which one to use and when? Front Immunol (2014) 5:105. doi: 10.3389/fimmu.2014.00105 24678311 PMC3958761

[42] Loh Z Simpson J Ullah A Zhang V Gan WJ Lynch JP. HMGB1 amplifies ILC2-induced type-2 inflammation and airway smooth muscle remodelling. PloS Pathog (2020) 16:e1008651. doi: 10.1371/journal.ppat.1008651 32658914 PMC7377495

[43] Narumi K Miyakawa R Ueda R Hashimoto H Yamamoto Y Yoshida T. Proinflammatory proteins S100A8/S100A9 activate NK cells *via* interaction with RAGE. J Immunol (2015) 194:5539–48. doi: 10.4049/jimmunol.1402301 25911757

[44] Souza-Fonseca-Guimaraes F Parlato M Philippart F Misset B Cavaillon J-M Adib-Conquy M. Toll-like receptors expression and interferon-γ production by NK cells in human sepsis. Crit Care (2012) 16:R206. doi: 10.1186/cc11838 23098236 PMC3682310

[45] Ishii T Muroi M Horiguchi K Tanamoto K-I Nagase T Yamashita N. Activation through toll-like receptor 2 on group 2 innate lymphoid cells can induce asthmatic characteristics. Clin Exp Allergy (2019) 49:1624–32. doi: 10.1111/cea.13490 31494992

[46] Hardman CS Chen Y-L Salimi M Nahler J Corridoni D Jagielowicz M. IL-6 effector function of group 2 innate lymphoid cells (ILC2) is NOD2 dependent. Sci Immunol (2021) 6. doi: 10.1126/sciimmunol.abe5084 PMC7611333 34021026

[47] Szomolanyi-Tsuda E Liang X Welsh RM Kurt-Jones EA Finberg RW. Role for TLR2 in NK cell-mediated control of murine cytomegalovirus *in vivo* . J Virol (2006) 80:4286–91. doi: 10.1128/JVI.80.9.4286-4291.2006 PMC1472014 16611887

[48] Cruz-Zárate D Cabrera-Rivera GL Ruiz-Sánchez BP Serafín-López J Chacón-Salinas R López-Macías C. Innate lymphoid cells have decreased HLA-DR expression but retain their responsiveness to TLR ligands during sepsis. J Immunol (2018) 201:3401–10. doi: 10.4049/jimmunol.1800735 30373848

[49] Sawaki J Tsutsui H Hayashi N Yasuda K Akira S Tanizawa T. Type 1 cytokine/chemokine production by mouse NK cells following activation of their TLR/MyD88-mediated pathways. Int Immunol (2007) 19:311–20. doi: 10.1093/intimm/dxl148 17289654

[50] Björklund ÅK Forkel M Picelli S Konya V Theorell J Friberg D. The heterogeneity of human CD127+ innate lymphoid cells revealed by single-cell RNA sequencing. Nat Immunol (2016) 17:451–60. doi: 10.1038/ni.3368 26878113

[51] Hazenberg MD Haverkate NJE van Lier YF Spits H Krabbendam L Bemelman WA. Human ectoenzyme-expressing ILC3: immunosuppressive innate cells that are depleted in graft-versus-host disease. Blood Adv (2019) 3:3650–60. doi: 10.1182/bloodadvances.2019000176 PMC6880892 31751473

[52] Li Z Gao Y He C Wei H Zhang J Zhang H. Purinergic receptor P2Y 6 is a negative regulator of NK cell maturation and function. J Immunol (2021) 207:1555–65. doi: 10.4049/jimmunol.2000750 34426542

[53] Kornum BR Kawashima M Faraco J Lin L Rico TJ Hesselson S. Common variants in P2RY11 are associated with narcolepsy. Nat Genet (2011) 43:66–71. doi: 10.1038/ng.734 21170044 PMC3019286

[54] Kudira R Malinka T Kohler A Dosch M de Agüero MG Melin N. P2X1-regulated IL-22 secretion by innate lymphoid cells is required for efficient liver regeneration. Hepatology (2016) 63:2004–17. doi: 10.1002/hep.28492 26853442

[55] Nabekura T Riggan L Hildreth AD O’Sullivan TE Shibuya A. Type 1 innate lymphoid cells protect mice from acute liver injury *via* interferon-γ secretion for upregulating bcl-xL expression in hepatocytes. Immunity (2020) 52:96–108.e9. doi: 10.1016/j.immuni.2019.11.004 31810881 PMC8108607

[56] Gu BJ Zhang WY Bendall LJ Chessell IP Buell GN Wiley JS. Expression of P2X 7 purinoceptors on human lymphocytes and monocytes: evidence for nonfunctional P2X 7 receptors. Am J Physiol Physiol (2000) 279:C1189–97. doi: 10.1152/ajpcell.2000.279.4.C1189 11003599

[57] Bourgeois E Van LP Samson M Diem S Barra A Roga S. The pro-Th2 cytokine IL-33 directly interacts with invariant NKT and NK cells to induce IFN-gamma production. Eur J Immunol (2009) 39:1046–55. doi: 10.1002/eji.200838575 19266498

[58] Smithgall MD Comeau MR Yoon B-RP Kaufman D Armitage R Smith DE. IL-33 amplifies both Th1- and Th2-type responses through its activity on human basophils, allergen-reactive Th2 cells, iNKT and NK cells. Int Immunol (2008) 20:1019–30. doi: 10.1093/intimm/dxn060 18550585

[59] Neill DR Wong SH Bellosi A Flynn RJ Daly M Langford TKA. Nuocytes represent a new innate effector leukocyte that mediates type-2 immunity. Nature (2010) 464:1367–70. doi: 10.1038/nature08900 PMC2862165 20200518

[60] Monticelli LA Sonnenberg GF Abt MC Alenghat T Ziegler CGK Doering TA. Innate lymphoid cells promote lung-tissue homeostasis after infection with influenza virus. Nat Immunol (2011) 12:1045–54. doi: 10.1031/ni.2131 PMC3320042 21946417

[61] Mathä L Martinez-Gonzalez I Steer CA Takei F. The fate of activated group 2 innate lymphoid cells. Front Immunol (2021) 12:671966. doi: 10.3389/fimmu.2021.671966 33968080 PMC8100346

[62] Hyodo Y Matsui K Hayashi N Tsutsui H Kashiwamura S Yamauchi H. IL-18 up-regulates perforin-mediated NK activity without increasing perforin messenger RNA expression by binding to constitutively expressed IL-18 receptor. J Immunol (1999) 162:1662–8. 9973427

[63] Silver JS Kearley J Copenhaver AM Sanden C Mori M Yu L. Inflammatory triggers associated with exacerbations of COPD orchestrate plasticity of group 2 innate lymphoid cells in the lungs. Nat Immunol (2016) 17:626–35. doi: 10.1038/ni.3443 PMC5345745 27111143

[64] Wang KS Frank DA Ritz J. Interleukin-2 enhances the response of natural killer cells to interleukin-12 through up-regulation of the interleukin-12 receptor and STAT4. Blood (2000) 95:3183–90. doi: 10.1182/blood.V95.10.3183 10807786

[65] Lim AI Menegatti S Bustamante J Le Bourhis L Allez M Rogge L. IL-12 drives functional plasticity of human group 2 innate lymphoid cells. J Exp Med (2016) 213:569–83. doi: 10.1084/jem.20151750 PMC4821648 26976630

[66] Robinette ML Fuchs A Cortez VS Lee JS Wang Y Durum SK. Transcriptional programs define molecular characteristics of innate lymphoid cell classes and subsets. Nat Immunol (2015) 16:306–17. doi: 10.1038/ni.3094 PMC4372143 25621825

[67] Fuchs A Vermi W Lee JS Lonardi S Gilfillan S Newberry RD. Intraepithelial type 1 innate lymphoid cells are a unique subset of IL-12- and IL-15-responsive IFN-γ-producing cells. Immunity (2013) 38:769–81. doi: 10.1016/j.immuni.2013.02.010 PMC3634355 23453631

[68] Yu H Fehniger TA Fuchshuber P Thiel KS Vivier E Carson WE. Flt3 ligand promotes the generation of a distinct CD34+Human natural killer cell progenitor that responds to interleukin-15. Blood (1998) 92:3647–57. doi: 10.1182/blood.V92.10.3647 9808558

[69] Rosmaraki EE Douagi I Roth C Colucci F Cumano A Di Santo JP. Identification of committed NK cell progenitors in adult murine bone marrow. Eur J Immunol (2001) 31:1900–9. doi: 10.1002/1521-4141(200106)31:6<1900::AID-IMMU1900>3.0.CO;2-M 11433387

[70] Robinette ML Bando JK Song W Ulland TK Gilfillan S Colonna M. IL-15 sustains IL-7R-independent ILC2 and ILC3 development. Nat Commun (2017) 8:14601. doi: 10.1038/ncomms14601 28361874 PMC5380969

[71] Simoni Y Fehlings M Kløverpris HN McGovern N Koo S-L Loh CY. Human innate lymphoid cell subsets possess tissue-type based heterogeneity in phenotype and frequency. Immunity (2017) 46:148–61. doi: 10.1016/j.immuni.2016.11.005 PMC7612935 27986455

[72] Weizman O-E Song E Adams NM Hildreth AD Riggan L Krishna C. Mouse cytomegalovirus-experienced ILC1s acquire a memory response dependent on the viral glycoprotein m12. Nat Immunol (2019) 20:1004–11. doi: 10.1038/s41590-019-0430-1 PMC6697419 31263280

[73] Ziblat A Nuñez SY Raffo Iraolagoitia XL Spallanzani RG Torres NI Sierra JM. Interleukin (IL)-23 stimulates IFN-γ secretion by CD56bright natural killer cells and enhances IL-18-Driven dendritic cells activation. Front Immunol (2017) 8:1959. doi: 10.3389/fimmu.2017.01959 29403472 PMC5785728

[74] Chen R Kang R Tang D. The mechanism of HMGB1 secretion and release. Exp Mol Med (2022) 54:91–102. doi: 10.1038/s12276-022-00736-w 35217834 PMC8894452

[75] Galluzzi L Buqué A Kepp O Zitvogel L Kroemer G. Immunogenic cell death in cancer and infectious disease. Nat Rev Immunol (2017) 17:97–111. doi: 10.1038/nri.2016.107 27748397

[76] Hernandez C Huebener P Schwabe RF. Damage-associated molecular patterns in cancer: a double-edged sword. Oncogene (2016) 35:5931–41. doi: 10.1038/onc.2016.104 PMC5119456 27086930

[77] Cheng B-Q Jia C-Q Liu C-T Lu X-F Zhong N Zhang Z-L. Serum high mobility group box chromosomal protein 1 is associated with clinicopathologic features in patients with hepatocellular carcinoma. Dig Liver Dis (2008) 40:446–52. doi: 10.1016/j.dld.2007.11.024 18294942

[78] Chung HW Lee S-G Kim H Hong DJ Chung JB Stroncek D. Serum high mobility group box-1 (HMGB1) is closely associated with the clinical and pathologic features of gastric cancer. J Transl Med (2009) 7:38. doi: 10.1186/1479-5876-7-38 19476625 PMC2694170

[79] Gebhardt C Riehl A Durchdewald M Németh J Fürstenberger G Müller-Decker K. RAGE signaling sustains inflammation and promotes tumor development. J Exp Med (2008) 205:275–85. doi: 10.1084/jem.20070679 PMC2271015 18208974

[80] Pusterla T Nèmeth J Stein I Wiechert L Knigin D Marhenke S. Receptor for advanced glycation endproducts (RAGE) is a key regulator of oval cell activation and inflammation-associated liver carcinogenesis in mice. Hepatology (2013) 58:363–73. doi: 10.1002/hep.26395 23504974

[81] Demoulin S Herfs M Somja J Roncarati P Delvenne P Hubert P. HMGB1 secretion during cervical carcinogenesis promotes the acquisition of a tolerogenic functionality by plasmacytoid dendritic cells. Int J Cancer (2015) 137:345–58. doi: 10.1002/ijc.29389 25492101

[82] Guerriero JL Ditsworth D Catanzaro JM Sabino G Furie MB Kew RR. DNA Alkylating therapy induces tumor regression through an HMGB1-mediated activation of innate immunity. J Immunol (2011) 186:3517–26. doi: 10.4049/jimmunol.1003267 PMC3066027 21300822

[83] Zhang K Jin Y Lai D Wang J Wang Y Wu X. RAGE-induced ILC2 expansion in acute lung injury due to haemorrhagic shock. Thorax (2020) 75:209–19. doi: 10.1136/thoraxjnl-2019-213613 PMC7063398 31937554

[84] Vijayan D Young A Teng MWL Smyth MJ. Targeting immunosuppressive adenosine in cancer. Nat Rev Cancer (2017) 17:709–24. doi: 10.1038/nrc.2017.86 29059149

[85] Di Virgilio F Sarti AC Falzoni S De Marchi E Adinolfi E. Extracellular ATP and P2 purinergic signalling in the tumour microenvironment. Nat Rev Cancer (2018) 18:601–18. doi: 10.1038/s41568-018-0037-0 30006588

[86] Hofman P Cherfils-Vicini J Bazin M Ilie M Juhel T Hébuterne X. Genetic and pharmacological inactivation of the purinergic P2RX7 receptor dampens inflammation but increases tumor incidence in a mouse model of colitis-associated cancer. Cancer Res (2015) 75:835–45. doi: 10.1158/0008-5472.CAN-14-1778 25564520

[87] Bernardazzi C Castelo-Branco MTL Pêgo B Ribeiro BE Rosas SLB Santana PT. The P2X7 receptor promotes colorectal inflammation and tumorigenesis by modulating gut microbiota and the inflammasome. Int J Mol Sci (2022) 23:4616. doi: 10.3390/ijms23094616 35563010 PMC9099551

[88] Crittenden S Cheyne A Adams A Forster T Robb CT Felton J. Purine metabolism controls innate lymphoid cell function and protects against intestinal injury. Immunol Cell Biol (2018) 96:1049–59. doi: 10.1111/imcb.12167 PMC6248310 29758102

[89] Liew FY Girard J-P Turnquist HR. Interleukin-33 in health and disease. Nat Rev Immunol (2016) 16:676–89. doi: 10.1038/nri.2016.95 27640624

[90] Griesenauer B Paczesny S. The ST2/IL-33 axis in immune cells during inflammatory diseases. Front Immunol (2017) 8:475. doi: 10.3389/fimmu.2017.00475 28484466 PMC5402045

[91] Choi M-R Sosman JA Zhang B. The janus face of IL-33 signaling in tumor development and immune escape. Cancers (Basel) (2021) 13:3281. doi: 10.3390/cancers13133281 34209038 PMC8268428

[92] Cui G Yuan A Pang Z Zheng W Li Z Goll R. Contribution of IL-33 to the pathogenesis of colorectal cancer. Front Oncol (2018) 8:561. doi: 10.3389/fonc.2018.00561 30547011 PMC6279916

[93] Eissmann MF Dijkstra C Jarnicki A Phesse T Brunnberg J Poh AR. IL-33-mediated mast cell activation promotes gastric cancer through macrophage mobilization. Nat Commun (2019) 10:2735. doi: 10.1038/s41467-019-10676-1 31227713 PMC6588585

[94] Eissmann MF Dijkstra C Wouters MA Baloyan D Mouradov D Nguyen PM. Interleukin 33 signaling restrains sporadic colon cancer in an interferon-γ–dependent manner. Cancer Immunol Res (2018) 6:409–21. doi: 10.1158/2326-6066.CIR-17-0218 29463593

[95] Cui G Qi H Gundersen MD Yang H Christiansen I Sørbye SW. Dynamics of the IL-33/ST2 network in the progression of human colorectal adenoma to sporadic colorectal cancer. Cancer Immunol Immunother (2015) 64:181–90. doi: 10.1007/s00262-014-1624-x PMC11028541 25324197

[96] McGeachy MJ Cua DJ Gaffen SL. The IL-17 family of cytokines in health and disease. Immunity (2019) 50:892–906. doi: 10.1016/j.immuni.2019.03.021 30995505 PMC6474359

[97] Reynolds JM Lee Y-H Shi Y Wang X Angkasekwinai P Nallaparaju KC. Interleukin-17B antagonizes interleukin-25-Mediated mucosal inflammation. Immunity (2015) 42:692–703. doi: 10.1016/j.immuni.2015.03.008 25888259 PMC5811222

[98] Thelen TD Green RM Ziegler SF. Acute blockade of IL-25 in a colitis associated colon cancer model leads to increased tumor burden. Sci Rep (2016) 6:25643. doi: 10.1038/srep25643 27165713 PMC4863374

[99] Dranoff G. Cytokines in cancer pathogenesis and cancer therapy. Nat Rev Cancer (2004) 4:11–22. doi: 10.1038/nrc1252 14708024

[100] Woodford D Johnson SD De Costa A-MA Young MRI. An inflammatory cytokine milieu is prominent in premalignant oral lesions, but subsides when lesions progress to squamous cell carcinoma. J Clin Cell Immunol (2014) 5:230. doi: 10.4172/2155-9899.1000230 25419481 PMC4240319

[101] Johnson SD De Costa A-MA Young MRI. Effect of the premalignant and tumor microenvironment on immune cell cytokine production in head and neck cancer. Cancers (Basel) (2014) 6:756–70. doi: 10.3390/cancers6020756 PMC4074802 24698959

[102] Santana Carrero RM Beceren-Braun F Rivas SC Hegde SM Gangadharan A Plote D. IL-15 is a component of the inflammatory milieu in the tumor microenvironment promoting antitumor responses. Proc Natl Acad Sci (2019) 116:599–608. doi: 10.1073/pnas.1814642116 30587590 PMC6329954

[103] Mascaux C Angelova M Vasaturo A Beane J Hijazi K Anthoine G. Immune evasion before tumour invasion in early lung squamous carcinogenesis. Nature (2019) 571:570–5. doi: 10.1038/s41586-019-1330-0 31243362

[104] Guia S Narni-Mancinelli E. Helper-like innate lymphoid cells in humans and mice. Trends Immunol (2020) 41:436–52. doi: 10.1016/j.it.2020.03.002 32223931

[105] Tugues S Burkhard SH Ohs I Vrohlings M Nussbaum K Vom Berg J. New insights into IL-12-mediated tumor suppression. Cell Death Differ (2015) 22:237–46. doi: 10.1038/cdd.2014.134 PMC4291488 25190142

[106] Vignali DAA Kuchroo VK. IL-12 family cytokines: immunological playmakers. Nat Immunol (2012) 13:722–8. doi: 10.1038/ni.2366 PMC4158817 22814351

[107] Cao X Leonard K Collins LI Cai SF Mayer JC Payton JE. Interleukin 12 stimulates IFN- -mediated inhibition of tumor-induced regulatory T-cell proliferation and enhances tumor clearance. Cancer Res (2009) 69:8700–9. doi: 10.1158/0008-5472.CAN-09-1145 PMC2783758 19843867

[108] Zhao J Zhao J Perlman S. Differential effects of IL-12 on tregs and non-treg T cells: Roles of IFN-γ, IL-2 and IL-2R. PLoS One (2012) 7:e46241. doi: 10.1371/journal.pone.0046241 23029447 PMC3459844

[109] Meeran SM Mantena SK Meleth S Elmets CA Katiyar SK. Interleukin-12-deficient mice are at greater risk of UV radiation-induced skin tumors and malignant transformation of papillomas to carcinomas. Mol Cancer Ther (2006) 5:825–32. doi: 10.1158/1535-7163.MCT-06-0003 16648552

[110] Liu J Xiang Z Ma X. Role of IFN regulatory factor-1 and IL-12 in immunological resistance to pathogenesis of n-methyl-N-nitrosourea-induced T lymphoma. J Immunol (2004) 173:1184–93. doi: 10.4049/jimmunol.173.2.1184 15240709

[111] Smyth MJ Taniguchi M Street SE. The anti-tumor activity of IL-12: mechanisms of innate immunity that are model and dose dependent. J Immunol (2000) 165:2665–70. doi: 10.4049/jimmunol.165.5.2665 10946296

[112] Langowski JL Zhang X Wu L Mattson JD Chen T Smith K. IL-23 promotes tumour incidence and growth. Nature (2006) 442:461–5. doi: 10.1038/nature04808 16688182

[113] Zheng Y Wang M Tian T Liu K Liu X Zhai Y. Role of interleukin-12 gene polymorphisms in the onset risk of cancer: a meta-analysis. Oncotarget (2017) 8:29795–807. doi: 10.18632/oncotarget.16080 PMC5444704 28415696

[114] Colonna M. Innate lymphoid cells: Diversity, plasticity, and unique functions in immunity. Immunity (2018) 48:1104–17. doi: 10.1016/j.immuni.2018.05.013 PMC6344351 29924976

[115] Mishra A Sullivan L Caligiuri MA. Molecular pathways: Interleukin-15 signaling in health and in cancer. Clin Cancer Res (2014) 20:2044–50. doi: 10.1158/1078-0432.CCR-12-3603 PMC3989546 24737791

[116] Kennedy MK Glaccum M Brown SN Butz EA Viney JL Embers M. Reversible defects in natural killer and memory CD8 T cell lineages in interleukin 15-deficient mice. J Exp Med (2000) 191:771–80. doi: 10.1084/jem.191.5.771 PMC2195858 10704459

[117] Klebanoff CA Finkelstein SE Surman DR Lichtman MK Gattinoni L Theoret MR. IL-15 enhances the *in vivo* antitumor activity of tumor-reactive CD8+ T cells. Proc Natl Acad Sci U S A (2004) 101:1969–74. doi: 10.1073/pnas.0307298101 PMC357036 14762166

[118] Szczepanski MJ Szajnik M Welsh A Foon KA Whiteside TL Boyiadzis M. Interleukin-15 enhances natural killer cell cytotoxicity in patients with acute myeloid leukemia by upregulating the activating NK cell receptors. Cancer Immunol Immunother (2010) 59:73–9. doi: 10.1007/s00262-009-0724-5 PMC3721322 19526239

[119] Kobayashi H Dubois S Sato N Sabzevari H Sakai Y Waldmann TA. Role of trans-cellular IL-15 presentation in the activation of NK cell-mediated killing, which leads to enhanced tumor immunosurveillance. Blood (2005) 105:721–7. doi: 10.1182/blood-2003-12-4187 15367431

[120] Huntington ND Alves NL Legrand N Lim A Strick-Marchand H Mention J-J. IL-15 transpresentation promotes both human T-cell reconstitution and t-cell–dependent antibody responses *in vivo* . Proc Natl Acad Sci (2011) 108:6217–22. doi: 10.1073/pnas.1019167108 PMC3076818 21444793

[121] Waldmann TA Dubois S Miljkovic MD Conlon KC. IL-15 in the combination immunotherapy of cancer. Front Immunol (2020) 11:868. doi: 10.3389/fimmu.2020.00868 32508818 PMC7248178

[122] Zhang C Zhang J Niu J Zhang J Tian Z. Interleukin-15 improves cytotoxicity of natural killer cells *via* up-regulating NKG2D and cytotoxic effector molecule expression as well as STAT1 and ERK1/2 phosphorylation. Cytokine (2008) 42:128–36. doi: 10.1016/j.cyto.2008.01.003 18280748

[123] Bahri R Pateras IS D’Orlando O Goyeneche-Patino DA Campbell M Polansky JK. IL-15 suppresses colitis-associated colon carcinogenesis by inducing antitumor immunity. Oncoimmunology (2015) 4:e1002721. doi: 10.1080/2162402X.2014.1002721 26405589 PMC4570106

[124] Gillgrass AE Chew MV Krneta T Ashkar AA. Overexpression of IL-15 promotes tumor destruction *via* NK1.1+ cells in a spontaneous breast cancer model. BMC Cancer (2015) 15:293. doi: 10.1186/s12885-015-1264-3 25879689 PMC4428091

[125] Park SL Buzzai A Rautela J Hor JL Hochheiser K Effern M. Tissue-resident memory CD8+ T cells promote melanoma–immune equilibrium in skin. Nature (2019) 565:366–71. doi: 10.1038/s41586-018-0812-9 30598548

[126] Mlecnik B Bindea G Angell HK Sasso MS Obenauf AC Fredriksen T. Functional network pipeline reveals genetic determinants associated with in situ lymphocyte proliferation and survival of cancer patients. Sci Transl Med (2014) 6:228ra37. doi: 10.1126/scitranslmed.3007240 24648340

[127] Klose CSN Flach M Möhle L Rogell L Hoyler T Ebert K. Differentiation of type 1 ILCs from a common progenitor to all helper-like innate lymphoid cell lineages. Cell (2014) 157:340–56. doi: 10.1016/j.cell.2014.03.030 24725403

[128] Nixon BG Chou C Krishna C Dadi S Michel AO Cornish AE. Cytotoxic granzyme c-expressing ILC1s contribute to antitumor immunity and neonatal autoimmunity. Sci Immunol (2022) 7:eabi8642. doi: 10.1126/sciimmunol.abi8642 35394814 PMC9233921

[129] Kansler ER Dadi S Krishna C Nixon BG Stamatiades EG Liu M. Cytotoxic innate lymphoid cells sense cancer cell-expressed interleukin-15 to suppress human and murine malignancies. Nat Immunol (2022) 23:904–15. doi: 10.1038/s41590-022-01213-2 PMC9202504 35618834

[130] Raykova A Carrega P Lehmann FM Ivanek R Landtwing V Quast I. Interleukins 12 and 15 induce cytotoxicity and early NK-cell differentiation in type 3 innate lymphoid cells. Blood Adv (2017) 1:2679–91. doi: 10.1182/bloodadvances.2017008839 PMC5745129 29296921

[131] Dinarello CA Novick D Kim S Kaplanski G. Interleukin-18 and IL-18 binding protein. Front Immunol (2013) 4:289. doi: 10.3389/fimmu.2013.00289 24115947 PMC3792554

[132] Tsutsui H Nakanishi K Matsui K Higashino K Okamura H Miyazawa Y. IFN-gamma-inducing factor up-regulates fas ligand-mediated cytotoxic activity of murine natural killer cell clones. J Immunol (1996) 157:3967–73. 8892629

[133] Dao T Ohashi K Kayano T Kurimoto M Okamura H. Interferon-gamma-inducing factor, a novel cytokine, enhances fas ligand-mediated cytotoxicity of murine T helper 1 cells. Cell Immunol (1996) 173:230–5. doi: 10.1006/cimm.1996.0272 8912881

[134] Matamoros JA da Silva MIF de Moura PMMF Leitão M da CG Coimbra EC. Reduced expression of IL-1β and IL-18 proinflammatory interleukins increases the risk of developing cervical cancer. Asian Pac J Cancer Prev (2019) 20:2715–21. doi: 10.31557/APJCP.2019.20.9.2715 PMC6976845 31554368

[135] Teng MWL Bowman EP McElwee JJ Smyth MJ Casanova J-L Cooper AM. IL-12 and IL-23 cytokines: from discovery to targeted therapies for immune-mediated inflammatory diseases. Nat Med (2015) 21:719–29. doi: 10.1038/nm.3895 26121196

[136] Pickard JM Maurice CF Kinnebrew MA Abt MC Schenten D Golovkina TV. Rapid fucosylation of intestinal epithelium sustains host-commensal symbiosis in sickness. Nature (2014) 514:638–41. doi: 10.1038/nature13823 PMC4214913 25274297

[137] Teng MWL Andrews DM McLaughlin N von Scheidt B Ngiow SF Möller A. IL-23 suppresses innate immune response independently of IL-17A during carcinogenesis and metastasis. Proc Natl Acad Sci (2010) 107:8328–33. doi: 10.1073/pnas.1003251107 PMC2889517 20404142

[138] Nasti TH Cochran JB Vachhani RV McKay K Tsuruta Y Athar M. IL-23 inhibits melanoma development by augmenting DNA repair and modulating T cell subpopulations. J Immunol (2017) 198:950–61. doi: 10.4049/jimmunol.1601455 PMC5225020 28003381

[139] Caughron B Yang Y Young MRI. Role of IL-23 signaling in the progression of premalignant oral lesions to cancer. PLoS One (2018) 13:e0196034. doi: 10.1371/journal.pone.0196034 29664967 PMC5903614

[140] Lanier LL. NK cell recognition. Annu Rev Immunol (2005) 23:225–74. doi: 10.1146/annurev.immunol.23.021704.115526 15771571

[141] Bryceson YT March ME Ljunggren H-G Long EO. Activation, coactivation, and costimulation of resting human natural killer cells. Immunol Rev (2006) 214:73–91. doi: 10.1111/j.1600-065X.2006.00457.x 17100877 PMC3845883

[142] Raulet DH Vance RE. Self-tolerance of natural killer cells. Nat Rev Immunol (2006) 6:520–31. doi: 10.1038/nri1863 16799471

[143] Morvan MG Lanier LL. NK cells and cancer: you can teach innate cells new tricks. Nat Rev Cancer (2016) 16:7–19. doi: 10.1038/nrc.2015.5 26694935

[144] Chan CJ Smyth MJ Martinet L. Molecular mechanisms of natural killer cell activation in response to cellular stress. Cell Death Differ (2014) 21:5–14. doi: 10.1038/cdd.2013.26 23579243 PMC3857624

[145] Raulet DH. Roles of the NKG2D immunoreceptor and its ligands. Nat Rev Immunol (2003) 3:781–90. doi: 10.1038/nri1199 14523385

[146] Guerra N Tan YX Joncker NT Choy A Gallardo F Xiong N. NKG2D-deficient mice are defective in tumor surveillance in models of spontaneous malignancy. Immunity (2008) 28:571–80. doi: 10.1016/j.immuni.2008.02.016 PMC3528789 18394936

[147] Champsaur M Lanier LL. Effect of NKG2D ligand expression on host immune responses. Immunol Rev (2010) 235:267–85. doi: 10.1111/j.0105-2896.2010.00893.x PMC2885032 20536569

[148] Groh V Rhinehart R Secrist H Bauer S Grabstein KH Spies T. Broad tumor-associated expression and recognition by tumor-derived gamma delta T cells of MICA and MICB. Proc Natl Acad Sci U.S.A. (1999) 96:6879–84. doi: 10.1073/pnas.96.12.6879 PMC22010 10359807

[149] Diefenbach A Jamieson AM Liu SD Shastri N Raulet DH. Ligands for the murine NKG2D receptor: expression by tumor cells and activation of NK cells and macrophages. Nat Immunol (2000) 1:119–26. doi: 10.1038/77793 11248803

[150] Cerwenka A Bakker AB McClanahan T Wagner J Wu J Phillips JH. Retinoic acid early inducible genes define a ligand family for the activating NKG2D receptor in mice. Immunity (2000) 12:721–7. doi: 10.1016/S1074-7613(00)80222-8 10894171

[151] Diefenbach A Jensen ER Jamieson AM Raulet DH. Rae1 and H60 ligands of the NKG2D receptor stimulate tumour immunity. Nature (2001) 413:165–71. doi: 10.1038/35093109 PMC3900321 11557981

[152] Cerwenka A Baron JL Lanier LL. Ectopic expression of retinoic acid early inducible-1 gene (RAE-1) permits natural killer cell-mediated rejection of a MHC class I-bearing tumor. vivo Proc Natl Acad Sci U.S.A. (2001) 98:11521–6. doi: 10.1073/pnas.201238598 PMC58762 11562472

[153] Ortner D Tripp CH Komenda K Dubrac S Zelger B Hermann M. Langerhans cells and NK cells cooperate in the inhibition of chemical skin carcinogenesis. Oncoimmunology (2016) 6:e1260215. doi: 10.1080/2162402X.2016.1260215 28344868 PMC5353916

[154] Girardi M Oppenheim DE Steele CR Lewis JM Glusac E Filler R. Regulation of cutaneous malignancy by γδ T cells. Sci (80- ) (2001) 294:605–9. doi: 10.1126/science.1063916 11567106

[155] Cipolat S Hoste E Natsuga K Quist SR Watt FM. Epidermal barrier defects link atopic dermatitis with altered skin cancer susceptibility. Elife (2014) 3:e01888. doi: 10.7554/eLife.01888 24843010 PMC4007207

[156] Fuertes MB Rossi LE Peralta CM Cabrera HN Allevato MA Zwirner NW. Premalignant quiescent melanocytic nevi do not express the MHC class I chain-related protein a. Medicina (B Aires) (2011) 71:357–60. 21893449

[157] Xuan XY Zhang JF Hu GM Li QR Liu PP Du Y. Upregulated expression of NKG2D and its ligands give potential therapeutic targets for patients with thymoma. Cancer Gene Ther (2015) 22:368–74. doi: 10.1038/cgt.2015.29 26113176

[158] Bottino C Castriconi R Pende D Rivera P Nanni M Carnemolla B. Identification of PVR (CD155) and nectin-2 (CD112) as cell surface ligands for the human DNAM-1 (CD226) activating molecule. J Exp Med (2003) 198:557–67. doi: 10.1084/jem.20030788 PMC2194180 12913096

[159] Iguchi-Manaka A Kai H Yamashita Y Shibata K Tahara-Hanaoka S Honda S. Accelerated tumor growth in mice deficient in DNAM-1 receptor. J Exp Med (2008) 205:2959–64. doi: 10.1084/jem.20081611 PMC2605241 19029379

[160] Croxford JL Tang MLF Pan MF Huang CW Kamran N Phua CML. ATM-Dependent spontaneous regression of early eμ-myc–induced murine b-cell leukemia depends on natural killer and T cells. Blood (2013) 121:2512–21. doi: 10.1182/blood-2012-08-449025 PMC4260366 23349395

[161] Guillerey C Ferrari de Andrade L Vuckovic S Miles K Ngiow SF Yong MCR. Immunosurveillance and therapy of multiple myeloma are CD226 dependent. J Clin Invest (2015) 125:2077–89. doi: 10.1172/JCI77181 PMC4463191 25893601

[162] Masson D Jarry A Baury B Blanchardie P Laboisse C Lustenberger P. Overexpression of the CD155 gene in human colorectal carcinoma. Gut (2001) 49:236–40. doi: 10.1136/gut.49.2.236 PMC1728395 11454801

[163] Liang S Yang Z Li D Miao X Yang L Zou Q. The clinical and pathological significance of nectin-2 and DDX3 expression in pancreatic ductal adenocarcinomas. Dis Markers (2015) 2015:1–8. doi: 10.1155/2015/379568 PMC4534609 26294807

[164] Rethacker L Roelens M Bejar C Maubec E Moins-Teisserenc H Caignard A. Specific patterns of blood ILCs in metastatic melanoma patients and their modulations in response to immunotherapy. Cancers (Basel) (2021) 13:1446. doi: 10.3390/cancers13061446 33810032 PMC8004602

[165] Moretta A Bottino C Vitale M Pende D Cantoni C Mingari MC. Activating receptors and coreceptors involved in human natural killer cell-mediated cytolysis. Annu Rev Immunol (2001) 19:197–223. doi: 10.1146/annurev.immunol.19.1.197 11244035

[166] Barrow AD Martin CJ Colonna M. The natural cytotoxicity receptors in health and disease. Front Immunol (2019) 10:909. doi: 10.3389/fimmu.2019.00909 31134055 PMC6514059

[167] Trabanelli S Chevalier MF Martinez-Usatorre A Gomez-Cadena A Salomé B Lecciso M. Tumour-derived PGD2 and NKp30-B7H6 engagement drives an immunosuppressive ILC2-MDSC axis. Nat Commun (2017) 8:593. doi: 10.1038/s41467-017-00678-2 28928446 PMC5605498

[168] Salimi M Xue L Jolin H Hardman C Cousins DJ McKenzie ANJ. Group 2 innate lymphoid cells express functional NKp30 receptor inducing type 2 cytokine production. J Immunol (2016) 196:45–54. doi: 10.4049/jimmunol.1501102 26582946 PMC4913864

[169] Sheppard S Schuster IS Andoniou CE Cocita C Adejumo T Kung SKP. The murine natural cytotoxic receptor NKp46/NCR1 controls TRAIL protein expression in NK cells and ILC1s. Cell Rep (2018) 22:3385–92. doi: 10.1016/j.celrep.2018.03.023 PMC5896200 29590608

[170] Halfteck GG Elboim M Gur C Achdout H Ghadially H Mandelboim O. Enhanced *in vivo* growth of lymphoma tumors in the absence of the NK-activating receptor NKp46/NCR1. J Immunol (2009) 182:2221–30. doi: 10.4049/jimmunol.0801878 19201876

[171] Lakshmikanth T Burke S Ali TH Kimpfler S Ursini F Ruggeri L. NCRs and DNAM-1 mediate NK cell recognition and lysis of human and mouse melanoma cell lines *in vitro* and *in vivo* . J Clin Invest (2009) 119:1251–63. doi: 10.1172/JCI36022 PMC2673866 19349689

[172] Glasner A Ghadially H Gur C Stanietsky N Tsukerman P Enk J. Recognition and prevention of tumor metastasis by the NK receptor NKp46/NCR1. J Immunol (2012) 188:2509–15. doi: 10.4049/jimmunol.1102461 22308311

[173] Ben Merzoug L Marie S Satoh-Takayama N Lesjean S Albanesi M Luche H. Conditional ablation of NKp46 + cells using a novel Ncr1 greenCre mouse strain: NK cells are essential for protection against pulmonary B16 metastases. Eur J Immunol (2014) 44:3380–91. doi: 10.1002/eji.201444643 25142413

[174] Turchinovich G Ganter S Bärenwaldt A Finke D. NKp46 calibrates tumoricidal potential of type 1 innate lymphocytes by regulating TRAIL expression. J Immunol (2018) 200:3762–8. doi: 10.4049/jimmunol.1701333 29661825

[175] Brandt CS Baratin M Yi EC Kennedy J Gao Z Fox B. The B7 family member B7-H6 is a tumor cell ligand for the activating natural killer cell receptor NKp30 in humans. J Exp Med (2009) 206:1495–503. doi: 10.1084/jem.20090681 PMC2715080 19528259

[176] Pogge von Strandmann E Simhadri VR von Tresckow B Sasse S Reiners KS Hansen HP. Human leukocyte antigen-B-Associated transcript 3 is released from tumor cells and engages the NKp30 receptor on natural killer cells. Immunity (2007) 27:965–74. doi: 10.1016/j.immuni.2007.10.010 18055229

[177] Simhadri VR Reiners KS Hansen HP Topolar D Simhadri VL Nohroudi K. Dendritic cells release HLA-B-Associated transcript-3 positive exosomes to regulate natural killer function. PloS One (2008) 3:e3377. doi: 10.1371/journal.pone.0003377 18852879 PMC2566590

[178] Cagnano E Hershkovitz O Zilka A Bar-Ilan A Golder A Sion-Vardy N. Expression of ligands to NKp46 in benign and malignant melanocytes. J Invest Dermatol (2008) 128:972–9. doi: 10.1038/sj.jid.5701111 17972960

[179] Arnon TI Markel G Bar-Ilan A Hanna J Fima E Benchetrit F. Harnessing soluble NK cell killer receptors for the generation of novel cancer immune therapy. PloS One (2008) 3:e2150. doi: 10.1371/journal.pone.0002150 18478075 PMC2364651

[180] Gutierrez-Silerio GY Franco-Topete RA Haramati J Navarrete-Medina EM Gutierrez-Franco J Bueno-Topete MR. Positive staining of the immunoligand B7-H6 in abnormal/transformed keratinocytes consistently accompanies the progression of cervical cancer. BMC Immunol (2020) 21:9. doi: 10.1186/s12865-020-0341-9 32138659 PMC7059382

[181] Carrega P Loiacono F Di Carlo E Scaramuccia A Mora M Conte R. NCR(+)ILC3 concentrate in human lung cancer and associate with intratumoral lymphoid structures. Nat Commun (2015) 6:8280. doi: 10.1038/ncomms9280 26395069

[182] Krzywinska E Kantari-Mimoun C Kerdiles Y Sobecki M Isagawa T Gotthardt D. Loss of HIF-1α in natural killer cells inhibits tumour growth by stimulating non-productive angiogenesis. Nat Commun (2017) 8:1597. doi: 10.1038/s41467-017-01599-w 29150606 PMC5694012

[183] Wagner M Ealey KN Tetsu H Kiniwa T Motomura Y Moro K. Tumor-derived lactic acid contributes to the paucity of intratumoral ILC2s. Cell Rep (2020) 30:2743–57.e5. doi: 10.1016/j.celrep.2020.01.103 32101749

